# Contrasting Prognostic Roles of Stromal Periostin Expression and Immune Cells Infiltration in Colorectal Carcinoma

**DOI:** 10.31557/APJCP.2026.27.1.389

**Published:** 2026-01-22

**Authors:** Ghada Farghaly Abd-Elhamid, Moemen Mostafa Ahmed Hafez, Hanan Ahmed Mohammed Eltayeb, Noha Abd El Rahim Aboulhagag

**Affiliations:** 1 *Department of Pathology, Faculty of Medicine, Assiut University, Assiut, Egypt.*; 2 *Department of Medical Oncology, South Egypt Cancer Institute, Assiut University, Assiut, Egypt.*

**Keywords:** Colorectal carcinoma, tumor microenvironment, periostin, immune cells, prognosis

## Abstract

**Background and objectives:**

Colorectal carcinoma (CRC) is one of the most common causes of cancer-related deaths worldwide. The prognosis of CRC patients is variable even within the same cancer stage. The tumor microenvironment (TME) plays a crucial role in CRC, yet its prognostic value remains incompletely understood. We hypothesize that different components of TME can affect patient outcomes in multiple ways. In this study, we assessed the prognostic implications of the desmoplastic reaction (DR) and immune cell infiltration within the TME of CRC through morphological and immunohistochemical (IHC) evaluation.

**Methods:**

A retrospective cohort of 65 CRC patients was examined. The patterns of DR and the density of tumor infiltrating lymphocytes (TILs) were evaluated in Hematoxylin and Eosin (H&E)-stained sections. IHC was performed for periostin (*POSTN*), CD8, CD4, and CD68. Associations with clinicopathological parameters and overall survival (OS), both in the entire study group and in the subgroup treated with adjuvant-therapy were investigated. The relation between different components of TME was also assessed.

**Results:**

High stromal *POSTN* expression correlated significantly with poor prognosis and reduced OS (p=0.005). High density of TILs in H&E-stained slides, CD8⁺, CD4⁺, and combined (CD8+CD4) T-lymphocytes was associated with improved OS (p=0.03, 0.02, p=0.03, and <0.001, respectively), while a high density of CD68+ macrophages was linked to poor prognosis (p=0.006). The combined (CD8+CD4) T-lymphocytes score emerged as an independent prognostic factor for OS (HR=0.1, p<0.001), outperforming the other studied parameters.

**Conclusion:**

Stromal *POSTN* expression and immune cell infiltration, particularly combined (CD8+CD4), offer significant prognostic insights in CRC and may guide therapeutic decisions.

## Introduction

Colorectal carcinoma (CRC) represents a significant global health burden. It is considered the third most common malignancy and the second cause of cancer-related deaths worldwide. In 2022, CRC accounted for 9.6% of global cancer incidence and 9.3% of cancer deaths [1]. Despite the utility of tumor, node, metastasis (TNM) staging as a prognostic tool, clinical outcomes vary substantially among patients within the same stage, suggesting a need for additional prognostic markers [2].

Recent studies highlight the tumor microenvironment (TME) as a critical determinant of tumor progression and therapeutic response. The TME is composed of various stromal components, including cancer-associated fibroblasts (CAFs), tumor-infiltrating lymphocytes (TILs), tumor-associated macrophages (TAMs), and extracellular matrix (ECM) proteins, all of which interact dynamically with tumor cells [3].

The overgrowth of fibrous connective tissue around tumor cells is known as desmoplastic reaction (DR). This DR is formed of a stromal matrix in which cancer cells and the peri-tumoral stromal cells are embedded [4]. Periostin (*POSTN*), a secreted ECM protein, has gained attention in the last few years due to its overexpression in various cancers, including CRC. It is also supposed that *POSTN* is related to different patterns of DR, which in turn affects tumor aggressiveness [5].

Despite growing interest in the TME, the interaction between stromal desmoplasia and the immune landscape in CRC remains poorly understood. While both desmoplasia and immune infiltration have been independently associated with tumor progression and patient outcomes, few studies have explored their potential influence on each other [6]. 

This study aims to investigate whether the distinct components of the tumor microenvironment, specifically DR highlighted by *POSTN* expression, and immune cell infiltration including *CD4⁺, CD8⁺* T-lymphocytes, and *CD68⁺* macrophages, contribute differently to the clinical outcomes of CRC patients. By employing histomorphological assessment and immunohistochemistry (IHC), we aim to elucidate the prognostic relevance and potential interaction between stromal desmoplasia and immune cell density, thereby providing a deeper understanding of tumor–stroma–immune crosstalk in CRC.

## Materials and Methods

### Study Design and Case Selection

This is a retrospective study that included 65 CRC patients who underwent radical resection between January 2017 and December 2020 at Assiut University Hospital (AUH) and South Egypt Cancer Institute (SECI). Inclusion criteria included cases with adequate clinical, pathological, and follow-up data and available tissue blocks. Patients receiving neoadjuvant therapy were excluded. 

### Histopathological Evaluation

All hematoxylin and eosin (H&E)-stained slides were available for histological review. The tumor type and grade were evaluated according to the WHO classification of colorectal neoplasms (2019) and staged using AJCC 8th edition criteria (2017). The presence or absence of lymphovascular invasion (LVI), perineural invasion (PNI), and lymph node (LN) metastasis was re-evaluated. 

The DR was evaluated at the tumor invasive margin, which is defined as the region centered on the border separating the host tissue from the malignant tissue, with an extent of 1 mm [7]. Cases were categorized as having mature, intermediate, or immature DR based on the presence or absence of myxoid stroma and keloid-like collagen as follows [8]:

### Immature

presence of an amorphous myxoid stroma, which is basophilic or amphophilic, filling at least a microscopic field of a 40x objective lens. 

### Intermediate

absence of significant myxoid stroma and presence of keloid-like thick collagen bundles of hypocellular collagen with bright eosinophilic hyalinization that was typically seen in keloid scars. 

### Mature

fibrotic stroma composed of fine mature collagen fibers stratified into multilayers without the presence of keloid-like collagen or myxoid stroma. 

According to the International TILs Working Group (ITWG) guidelines, TILs were defined as the mononuclear immune cells (lymphocytes and plasma cells) within the tumor stroma [9]. The density of TILs was evaluated at the invasive margin as the percentage of the stromal area that was occupied by mononuclear immune cells in five random high power fields (HPFs) “Olympus Trinocular microscope CX33, 0.65 mm field diameter, 40x objective lens” and the mean value was calculated to represent the average TILs density for each case as described by Matsutani et al. [10]. For statistical analysis, cases were classified as high and low TILs groups based on a 30% cut-off value obtained by using X-tile software (version 3.6.1, Yale University, USA). 

### Immunohistochemistry and Marker Assessment

Immunohistochemistry was performed to assess *POSTN*,* CD8, CD4*, and *CD68* expression at the invasive tumor margin. The IHC technique was conducted according to the manufacturer’s protocols. Sections were incubated at room temperature for one hour with enough amount of primary antibodies of Anti-*POSTN* (1/200, Clone MD203, Catalog No. MC0373, Medaysis Company, USA), *CD68* (1/200, Clone Kp-1, Catalog No. CD246-02, Quartett Company, Germany), and CD8 (1/100, Clone C8/144B, Catalog No. CD039-02, Quartett Company, Germany). For CD4 staining, sections were incubated overnight at 4 °C with an adequate amount of Anti-CD4 antibody reagent (1/50, Clone QR032, Catalog No. CD024-02, Quartett Company, Germany). In each staining run, negative control slides were included to confirm the validity of staining. 

Slides of IHC-stained tissues were evaluated by two experienced pathologists (GF and NA), blinded to the clinicopathological data. *POSTN* was assessed in the tumor stroma. The intensity of immunostaining was scored as 0 (negative), 1 (weak), 2 (moderate), or 3 (strong), while the extent of staining was scored as 0 (0–4%), 1 (5–24%), 2 (25–49%), 3 (50–74%), or 4 (75–100%). The final score was calculated as the summation of staining and extent scores, and cases were classified as low *POSTN* expression group (total score, 0–4) or high group (total score, 5–7) as mentioned by Sueyama et al. [8]. 

The density of *CD8+, CD4+* T lymphocytes, and *CD68*+ TAMs was assessed by counting the immune-reactive cells in five random HPFs along the invasive margin. The mean value was calculated to represent the average density of each marker for each case as described previously [11, 12]. Sum of *CD4+* and *CD8+* T cells was calculated to detect the density of total T lymphocytes for each case. 

Counting of immune-reactive cells was conducted manually with the aid of ImageJ software program (version 1.51j8, Rasband W, National Institutes of Health, USA). Images were captured by an Olympus Digital Camera (EP50) connected to Olympus Trinocular microscope “CX33”. 

Cut-off values for each biomarker stratification were determined using X-tile software (version 3.6.1, Yale University, USA), and cases were classified as high and low groups.

### Survival data

Overall survival (OS) was measured as the interval between the date of diagnosis and the date of last clinic visit or death. Three years follow-up data were used for statistical analysis.

### Statistical Analysis

Categorical associations were analyzed via chi-square tests. Kaplan-Meier and log-rank tests were used for survival analyses. Cox regression test was used for multivariate analysis. p-values of less than 0.05 are considered statistically significant.

### Ethical approval

This study was approved by the Institutional Review Board of Assiut University, Assiut, Egypt (Approval No. 17200574).

## Results

### Clinicopathological Characteristics

The study cohort included 65 patients. All clinicopathological data of enrolled cases are shown in supplementary Table 1.

### Association of DR and H&E-assessed TILs with clinicopathological characteristics

**Figure 1:**
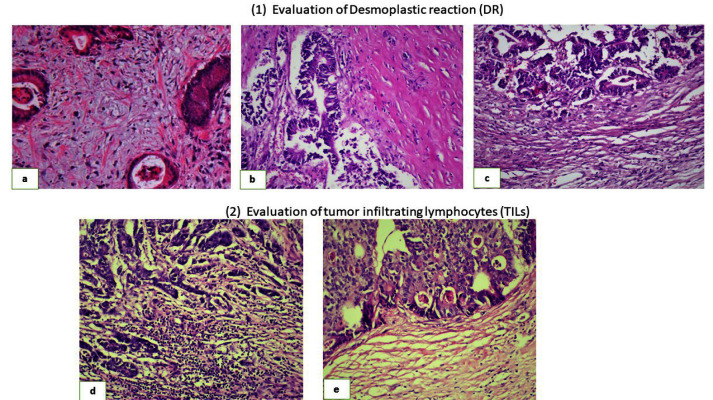
Evaluation of Different Patterns of DR: (a) immature, (b) intermediate, (c) mature, and the density of TILs: (d) high, (e) low (H&E, 20x objective lens)

Evaluation of DR using H&E-stained sections revealed that immature, intermediate and mature DR (Figure 1a, 1b, and 1c respectively) were present in 35.4%, 24.6%, and 40% of CRC, respectively. High and low TILs density (Figure 1d, and 1e respectively), were present in 66.2% and 33.8% of cases respectively.

Supplementary Table 2 demonstrates the relationship between different types of DR and the studied clinicopathological parameters. The data regarding the association between TILs density and the clinicopathological characteristics were summarized in supplementary Table 3. 

### Immunohistochemical analysis

#### Association of POSTN expression and clinicopathological characteristics

**Figure 2:**
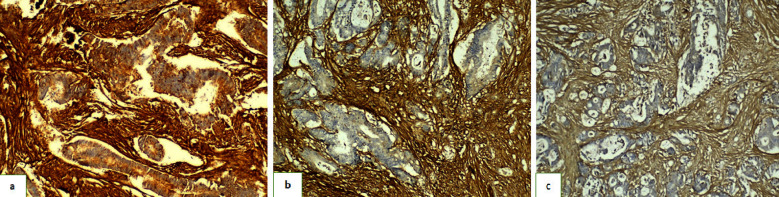
Stromal *POSTN* Expression with Variable Staining Intensity: (a) strong, (b) moderate, and (c) weak (IHC, 20x objective lens)

Stromal *POSTN* expression was assessed as regards the intensity of expression (strong, moderate, weak) “Figure 2” and the extent of staining. Cases were categorized as the high group (58.5%) and low group (41.5%). High stromal *POSTN* expression was significantly associated with high tumor grade (p=0.04), LVI (p=0.01), LN metastasis (p=0.005), and advanced pT stage (p<0.001) “Table 1”. 

### Association of immune cell infiltrate and clinicopathological characteristics

**Figure 3:**
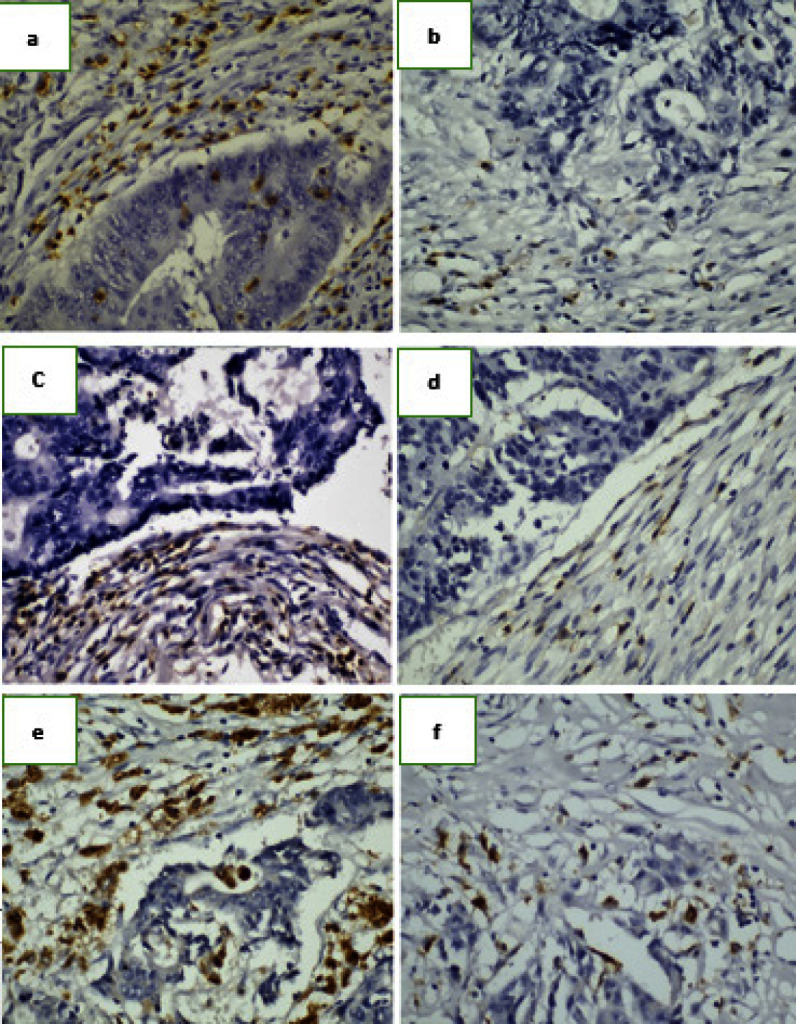
Evaluation of *CD8, CD4* and *CD68* Expression in the Tumor Stroma: (a) high *CD8* expression, (b) low* CD8 *expression, (c) high* CD4* expression, (d) low* CD4* expression, (e) high *CD68 *expression and (f) low *CD68* expression (IHC, 40x objective lens)

The expression of CD8, CD4, and *CD68* biomarkers was evaluated, and cases were classified as high and low groups (Figure 3). *CD8+* T-cells showed high expression in 67.7% of cases, while 36.9% of tumors had high *CD4+* T-cell density. Assessment of the total T lymphocytes density was calculated as the sum of *CD8+* and *CD4+* cells. It was found to be high in 61.5% of enrolled cases. High *CD68*+ TAMs constituted 67.7% of CRC patients. 

Significant associations were detected between IHC-assessed T lymphocytes and multiple clinicopathologic parameters (Table 2). High expression of CD8 was negatively associated with PNI (p=0.03). Less frequent LVI and LN metastases were shown in CRC displaying high CD4 expression (p=0.01 and 0.002, respectively). Assessment of the total T lymphocytes density (CD8 + CD4) revealed an association between high total T lymphocytes density and early-stage tumors “pT2” (p=0.004). Also, it showed inverse relation with the presence of LVI, PNI, and LN metastasis (p=0.01, 0.02, and 0.005, respectively). 

There was a significant association between high *CD68*+ TAMs and the presence of PNI (p=0.02), LVI (p=0.01), LN metastasis (p=0.05), and advanced tumor stage (p=0.01) “Table 2”.

### Association of POSTN expression and immune cell markers

High stromal *POSTN* expression was significantly associated with low CD4 expression and the low density of total (CD8 + CD4) T lymphocytes (p=0.009 and 0.02 respectively), while it showed significant relation with high *CD68* expression (p=0.005) “Supplementary Table 4”. Furthermore, a significant inverse association between *CD68* and CD4 expression was noticed (p=0.003) “Supplementary Table 5”.

### Survival Outcomes

**Figure 4:**
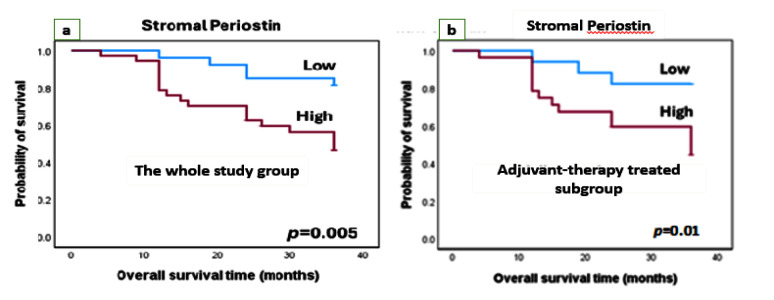
Kaplan Meier Survival Curves Show Significantly Shorter OS Associated with High rather than Low Stromal *POSTN* Expression in the whole Study Group (a), and adjuvnat-therapy treated subgroup (b)

Evaluation of DR in H&E-stained sections revealed an insignificant relation with the OS of the enrolled patients. However, using *POSTN* as IHC marker of the DR revealed that tumors exhibiting high stromal *POSTN* expression had significantly lower 3-year OS rates compared to those with low expression levels (p=0.005). Moreover, patients who received adjuvant therapy and had high *POSTN* expression showed poorer outcomes compared to those with low *POSTN* expression (p = 0.01) “Figure 4”. 

**Figure 5:**
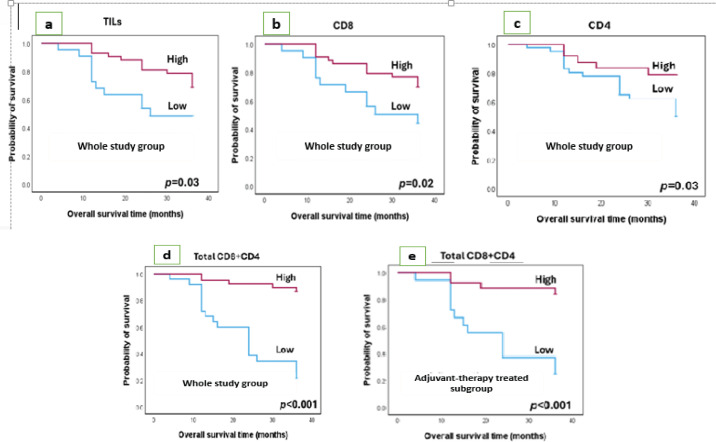
Kaplan Meier Survival Curves Show Prolonged OS in the whole Study Group with High Density of H&E-assessed TILs (a), high *CD8* (b), high *CD4* (c), high total (*CD8 + CD4*) expression (d), and better OS in patients received adjuvant therapy with high total (*CD8 + CD4*) expression (e)

Favorable OS was significantly observed in high vs low densities of H&E-assessed TILs, *CD8+*, *CD4+*, and total (*CD8+*CD4) T cells (p=0.03, 0.02, 0.03, and <0.001 respectively). Additionally, better OS was observed in adjuvant-therapy treated patients with high total (*CD8+*CD4) T cell densities rather than patients with low densities “Figure 5”. 

**Figure 6:**
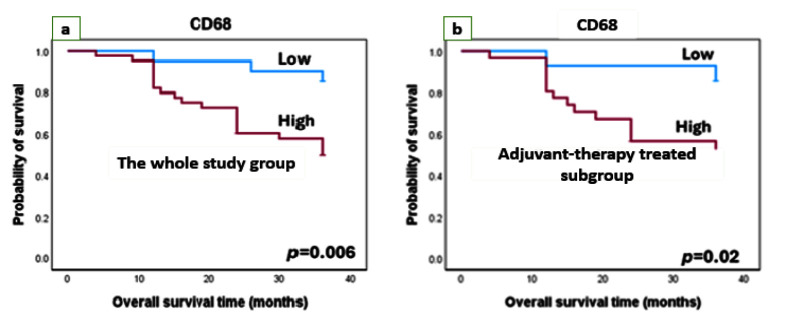
Kaplan Meier Survival Curves Show Significantly Shorter OS Associated with High Rather than Low *CD68 *Expression in the whole Study Group (a), and adjuvnat-therapy treated subgroup (b)

Regarding the prognostic role of TAMs, patients with high *CD68* expression had significantly shorter OS rates than those with low expression in the whole study group (p=0.006), and also in adjuvant-therapy treated subgroup (p=0.02) “Figure 6”.

A multivariate analysis model was employed to investigate the independent prognostic factors for OS among the different studied parameters (Table 3). The analysis revealed that the total (CD8 + CD4) T-lymphocytes count was the only independent predictor for OS in CRC patients (Hazard ratio = 0.1, 95% confidence interval = 0.02-0.3, p < 0.001). 

## Discussion

Despite the presence of different treatment modalities, CRC remains incurable in about 50% of cases, and treated patients show variable responses to the classic systemic therapy [13]. Over the past few years, the tumor-host interaction has gained attention as it is suggested to play an important role in tumor heterogeneity and subsequently affects patients’ outcome [6]. Recent studies stated that cancer development and progression are influenced by interactions between different components of TME, including tumor cells, immune cells, other stromal cells, and various components of ECM [14].

In this study, we evaluated the DR through histological assessment and IHC analysis of *POSTN* expression. The study revealed that, the immature DR “assessed morphologically and by *POSTN* staining” was significantly related to poor clinicopathological parameters, especially advanced tumor stage. Additionally, high stromal *POSTN* expression was significantly associated with high tumor grade, as well as the of presence LVI and LN metastasis. These findings were consistent with prior studies investigating *POSTN* as an ECM protein that activates the Akt pathway in CRC which promotes cell survival, invasion, and metastasis, suggesting *POSTN* as a potential prognostic biomarker in CRC [2, 8]. 

The prognostic value of immune cells infiltrate within the TME was previously studied, and it was found that high TILs density was associated with favorable histopathological features. The current study was in line with the previous reports as we found that, the high densities of H&E-assessed TILs and IHC-quantified T cells were significantly linked to multiple favorable histologic parameters, including early tumor stages, the less frequent LVI, PNI, and lymph node metastasis. These findings reflect the crucial role of T-cells in the host immune response against tumor progression [6, 15, 16].

The role of TAMs, by *CD68* staining, was evaluated in this study, where high expression was significantly associated with clinicopathological parameters of aggressive behavior. These findings were consistent with the study of Badawi et al., who reported that macrophage infiltration significantly increase in parallel with clinical stage and lymph node metastasis [17]. In contrast, Jedinak et al. demonstrated that increased macrophage infiltration was associated with improved outcomes in colon cancer [18]. This discrepancy may be attributed to differences in the predominant macrophage phenotype. In advanced tumors, TAMs tend to exhibit an M2-like profile, which is associated with a pro-tumorigenic activity through enhanced invasion, metastasis, and angiogenesis [12]. 

To the best of our knowledge, this is the first study that investigated the interplay between TME components: stromal and immune cells, highlighted by IHC. High stromal *POSTN* expression was significantly associated with low TILs density, which favors a potential role of *POSTN* in creating an immunosuppressive TME. It has been reported that, *POSTN* overexpression leads to activation of CAFs which in turn prevent the penetration of T lymphocytes within the tumor stroma [19]. 

In addition, there was a statistically significant association between high stromal *POSTN* and high *CD68* expression. It was previously mentioned that *POSTN* overexpression activates CAFs which secrete a group of cytokines as Interleukin 6 (*IL-6*). These cytokines increase the influx of monocytes into the TME as well as stimulate their differentiation to M2- macrophage phenotype [20].

Furthermore, we observed an inverse significant relation between *CD68* and CD4 expression. This may be explained by the immunosuppressive effects of M2 macrophages which are mediated the secretion of anti-inflammatory cytokines like *IL-10*, that impair T-cell function and contribute to immune evasion [21].

Survival analysis revealed that the DR assessed in H&E-stained sections showed no significant association with the OS. However, IHC assessment of DR (using anti-*POSTN* antibody) revealed a significant relation between high stromal *POSTN* expression and poor OS in the whole study group. Furthermore, patients who received adjuvant therapy and had high stromal *POSTN* expression showed worse OS than those with low stromal *POSTN* expression. This was consistent with previous studies that found an association between high *POSTN* expression and worse survival rates, including OS and disease free survival, and chemo-resistance in CRC patients [2, 22]. These findings signify the role of *POSTN* as a possible prognostic maker for risk stratification and as a predictive biomarker to guide treatment decisions regarding chemotherapy in CRC. 

The present study revealed a statistically significant association between high densities of H&E-assessed TILs, CD8+, *CD4+*, and total (CD8 + CD4) T lymphocytes and prolonged OS in the whole study group. This is in parallel with previous studies, which found that the density of H&E-assessed TILs and *CD8+* T-cell density at the invasive margin can predict prognosis in CRC patients [10, 15, 23]. Analysis of the relation between T-cell density and OS in the adjuvant-therapy treated subgroup showed a significant relation only between high total (CD8 + CD4) density and better OS. This suggests the possible synergistic effect and complementary roles of *CD8+* and *CD4+* T lymphocytes in predicting better survival of adjuvant therapy-treated patients. However, further studies on large cohorts are recommended to confirm these results. 

The present study revealed that high *CD68* expression was associated with poor OS in the whole study group and also in the adjuvant-therapy treated subgroup. This was in accordance with the data published in the literature about the predominance of M2-type TAMs in advanced tumor stages and their role in cancer progression and worse patients’ prognosis [17]. Also, this may indicate that patients with low *CD68* expression are more likely to benefit from adjuvant therapy, whereas those with high expression may not benefit as much. 

In conclusion, this study underscores the significance of stromal *POSTN* and immune cell components as prognostic biomarkers in CRC. Stromal *POSTN* expression has superior prognostic value over the morphologic assessment of DR in predicting patients’ outcome. High stromal *POSTN* and high *CD68* expressions correlate with poor OS in the whole study group and adjuvant-therapy treated subgroup, suggesting their potential use as prognostic and therapeutic targets. High density of stromal TILs and elevated CD8+ and *CD4+* T-cell densities predict favorable outcomes, supporting their role in tumor suppression and highlighting their prognostic value. The total (CD8 + CD4) T-cell density was the only independent predictive factor of OS among all studied parameters. Thus, the use of IHC-based quantification of T-cell subsets could be used as the most reliable approach for prognostication in CRC. The significant relation between different components of TME underscores the complex interactions between TME components and their combined influence on tumor progression and patients’ outcomes. 

Further studies are warranted to explore the utility of *POSTN* as a therapeutic target. Also, future studies including a larger sample size are recommended to study the link between TME components and specific molecular subtypes of CRC.

**Table 1:** Association of Stromal *POSTN* Expression and Clinicopathological Characteristics

Characteristics	High stromal POSTN (n=38) N (%)	Low stromal POSTN (n=27) N (%)	X^2^ (p-value)
Age at diagnosis (years)			
< 50	16 (42.1)	14 (51.9)	0.6 (0.4)
≥ 50	22 (57.9)	13 (48.1)	
Sex			
Male	15 (39.5)	14 (51.9)	0.9 (0.3)
Female	23 (60.5)	13 (48.1)	
Tumor site			
Right colon	17 (44.7)	14 (51.9)	0.7 (0.3)
Left colon	17 (44.7)	12 (44.4)	
Rectum	4 (10.6)	1 (3.7)	
Tumor size			
<5 cm	21 (55.2)	11 (40.7)	1.3 (0.2)
≥5 cm	17 (44.8)	16 (59.3)	
Pathological tumor stage			
pT2	1 (2.6)	16 (59.3)	23.1 (<0.001)*
pT3	27 (71.1)	10 (37)	
pT4	10 (26.3)	1 (3.7)	
Tumor grade			
Low grade	23 (60.5)	23 (85.2)	4.1 (0.04)*
High grade	15 (39.5)	4 (14.8)	
Lymph node metastasis			
Absent	12 (31.6)	18 (66.7)	57.8 (0.005)*
Present	26 (68.4)	9 (33.3)	
Lymphovascular invasion			
Absent	9 (23.7)	14 (51.9)	5.5 (0.01)*
Present	29 (76.3)	13 (48.1)	
Perineural invasion			
Absent	20 (52.6)	20 (74.1)	3.1 (0.08)
Present	18 (27.4)	7 (25.9)	
Histological subtype			
Adenocarcinoma, NOS	31 (81.6)	27 (100)	2.4 (0.1)
Mucinous adenocarcinoma	7 (18.4)	0 (0)	

* Statistically significant p-value.

**Table 2:** Association of* CD8, CD4,* total (*CD8 + CD4*), and* CD68* Expression and Clinicopathological Characteristics

Characteristics	High CD8 (n= 44) N (%)	Low CD8 (n= 21) N (%)	X^2^ (p-value)	High CD4 (n= 24) N (%)	Low CD4 (n= 41) N (%)	X^2^ (p-value)	High (CD8+CD4) (n= 40) N (%)	Low (CD8+CD4) (n= 25) N (%)	X^2^ (p-value)	High CD68 (n= 44) N (%)	Low CD68 (n= 21) N (%)	X^2^ (p-value)
Age at diagnosis (years)												
< 50	21 (47.7)	9 (42.9)	0.1 (0.7)	12 (50)	18 (43.9)	0.2 (0.6)	22 (55.0)	8 (32.0)	3.3 (0.07)	18 (40.9)	12 (57.1)	1.5 (0.2)
≥ 50	23 (52.3)	12 (57.1)		12 (50)	23 (56.1)		18 (45.0)	17 (68.0)		26 (59.1)	9 (42.9)	
Sex												
Male	21 (47.7)	8 (38.1)	0.5 (0.4)	9 (37.5)	20 (48.7)	0.7 (0.3)	19 (47.0)	10 (40.0)	0.4 (0.5)	20 (45.5)	9 (42.9)	0.03 (0.8)
Female	23 (52.3)	13 (61.9)		15 (62.5)	21 (51.3)		21 (53.0)	15 (60.0)		24 (54.5)	12 (57.1)	
Tumor site												
Right colon	22 (50)	9 (42.9)	1 (0.3)	12 (50)	19 (46.3)	0.9 (0.3)	18 (45.0)	13 (52.0)	0.1 (0.6)	19 (43.2)	12 (57.1)	1.2 (0.2)
Left colon	20 (45.5)	9 (42.9)		12 (50)	17 (41.5)		21 (52.0)	8 (32.0)		21 (47.7)	8 (38.1)	
Rectum	2 (4.5)	3 (14.2)		0	5 (12.2)		1 (3.0)	4 (16.0)		4 (9.1)	1 (4.8)	
Tumor size												
<5 cm	14 (32.0)	8 (38.0)	0.2 (0.6)	10 (41.6)	12 (29.2)	1 (0.3)	15 (37.0)	7 (28.0)	0.6 (0.4)	14 (32.0)	8 (38.0)	0.3 (0.6)
≥5 cm	30 (68.0)	13 (62.0)		14 (58.4)	29 (70.8)		25 (63.0)	18 (72.0)		30 (68.0)	13 (62.0)	
Pathological tumor stage												
pT2	14 (31.9)	3 (14.3)	2.5 (0.1)	9 (37.5)	8 (19.5)	3.5 (0.06)	15 (37.0)	2 (8.0)	8.1 (0.004)*	7 (15.9)	10 (47.6)	6 (0.01)*
pT3	24 (54.5)	13 (61.9)		13 (54.2)	24 (58.5)		21 (53.0)	16 (64.0)		28 (63.6)	9 (42.9)	
pT4	6 (13.6)	5 (23.8)		2 (8.3)	9 (22)		4 (10.0)	7 (28.0)		9 (20.5)	2 (9.5)	
Tumor grade												
Low grade	31 (70.5)	15 (71.4)	0.1 (0.7)	19 (79.2)	27 (65.9)	1 (0.3)	29 (73.0)	17 (68.0)	0.1 (0.6)	28 (65.0)	18 (86.0)	2.9 (0.08)
High grade	13 (29.5)	6 (28.6)		5 (20.8)	14 (29.3)		11 (27.0)	8 (32.0)		16 (35.0)	3 (14.0)	
Lymph node metastasis												
Absent	23 (52.3)	7 (33.3)	2.1 (0.1)	17 (70.8)	13 (31.7)	9.3 (0.002)*	24 (60.0)	6 (24.0)	8 (0.005)*	15 (34.1)	15 (71.4)	7.9 (0.005)*
Present	21 (47.7)	14 (66.7)		7 (29.2)	28 (68.3)		16 (40.0)	19 (76.0)		29 (65.9)	6 (28.6)	
Lymphovascular invasion												
Absent	18 (40.9)	5 (23.8)	1.8 (0.1)	13 (54.2)	10 (24.4)	5.9 (0.01)*	19 (47.0)	4 (16.0)	6.7 (0.01)*	11 (25.0)	12 (57.1)	6.4 (0.01)*
Present	26 (59.1)	16 (76.2)		11 (45.8)	31 (75.6)		21 (53.0)	21 (84.0)		33 (75.0)	9 (42.9)	
Perineural invasion												
Absent	31 (70.5)	9 (42.9)	4.6 (0.03)*	18 (75)	22 (53.7)	2.9 (0.8)	29 (73.0)	11 (44.0)	5.3 (0.02)*	23 (52.3)	17 (80.9)	4.9 (0.02)*
Present	13 (29.5)	12 (57.1)		6 (25)	19 (46.3)		11 (27.0)	14 (56.0)		21 (47.7)	4 (19.1)	
Histological subtype												
Adenocarcinoma, NOS	39 (88.6)	19 (90.5)	0.05 (0.8)	23 (95.8)	35 (85.4)	1.9 (0.1)	36 (90.0)	22 (88.0)	0.06 (0.8)	38 (86.4)	20 (95.2)	1.3 (0.2)
Mucinous adenocarcinoma	5 (11.4)	2 (9.5)		1 (4.2)	6 (14.6)		4 (10.0)	3 (12.0)		6 (13.6)	1 (4.8)	

* Statistically significant p-value.

**Table 3:** Multivariate Analysis Model Test of Stromal *POSTN* Expression and IHC-assessed Immune Markers as Independent Prognostic Factors for OS

Parameter	Hazard ratio	95% confidence interval	p-value
Stromal *POSTN*	0.4	0.2-2.5	0.1
*CD8*	1.6	0.7-3.9	0.2
*CD4*	0.9	0.3-3.1	0.9
Total *CD8+CD4*	0.1	0.02-0.3	<0.001*
*CD68*	0.4	0.1-1.5	0.2

* Statistically significant p-value.

## Author Contribution Statement

Conceptualization: All authors; Formal analysis: Ghada Farghlay Abd-Elhamid; Funding acquisition: Ghada Farghlay Abd-Elhamid, Moemen Mostafa Hafez, Noha Abdelrahim Abouelhagag; Investigation: Ghada Farghlay Abd-Elhamid, Moemen Mostafa Hafez, Noha Abdelrahim Abouelhagag; Methodology: Ghada Farghlay Abd-Elhamid, Moemen Mostafa Hafez, Noha Abdelrahim Abouelhagag; Resources: Ghada Farghlay Abd-Elhamid, Hanan Ahmed Eltayeb; Supervision: Moemen Mostafa Hafez, Noha Abdelrahim Abouelhagag, Hanan Ahmed Eltayeb; Writing – original draft: Ghada Farghlay Abd-Elhamid; Writing – review & editing: Moemen Mostafa Hafez, Noha Abdelrahim Abouelhagag, Hanan Ahmed Eltayeb; Approval of final manuscript: All authors.
